# Biomechanical Comparison of Patient-Specific Temporomandibular Joint Prostheses Using Ti6Al4V and CoCrMo Alloys: A Finite Element Analysis

**DOI:** 10.3390/ma18214822

**Published:** 2025-10-22

**Authors:** Ezgi Yüceer-Çetiner, Yasin Doğu, Hakan Yurten, Altan Varol

**Affiliations:** 1 Department of Oral and Maxillofacial Surgery, School of Dental Medicine, Bahcesehir University, Istanbul 34357, Turkey; altan.varol@bau.edu.tr; 2 Department of Research and Development, TST Medical Devices Co., Ltd., Istanbul 34906, Turkey; ar-ge@tstsan.com; 3 Department of Orthopedics and Traumatology, Fatsa Meditech Private Hospital, Ordu 52400, Turkey; hakanyurten@gmail.com

**Keywords:** biomechanics, finite element analysis, temporomandibular joint prosthesis, patient-specific implant, Ti6Al4V alloy, CoCrMo alloy

## Abstract

End-stage temporomandibular joint (TMJ) disorders often necessitate total joint replacement, and the selection of biomaterial directly impacts long-term outcomes. Ti6Al4V and CoCrMo are commonly used alloys, yet their biomechanical performance in patient-specific prostheses remains insufficiently compared. This study aimed to evaluate the mechanical response of custom TMJ prostheses fabricated from these alloys using finite element analysis (FEA). A three-dimensional mandibular model was created from computed tomography data, and a patient-specific prosthesis was designed in SolidWorks (Dassault Systèmes, SolidWorks Corp., Waltham, MA, USA) and analyzed in ANSYS Workbench 2022 R1 (Ansys Inc., Canonsburg, PA, USA). Physiological loading was simulated by applying forces at the insertion sites of the temporalis, masseter, and medial pterygoid muscles. In the Ti6Al4V model, maximum von Mises stresses reached 192.18 MPa on the mandibular component and 92.004 MPa on the fossa prosthesis, whereas the CoCrMo model demonstrated higher stresses of 204.31 MPa and 94.182 MPa, respectively. Both alloys exhibited similar stress distributions, but Ti6Al4V generated lower stress magnitudes, indicating more favorable load transfer and a reduced risk of mechanical overload on articulating components. These findings underscore the significance of alloy selection in optimizing TMJ prostheses and demonstrate the value of FEA as a tool for guiding future patient-specific designs.

## 1. Introduction

End-stage temporomandibular joint (TMJ) disorders such as severe degenerative changes, ankylosis, or post-traumatic condylar destruction often necessitate total joint replacement, particularly in patients with multiple failed surgical interventions [1,2]. These prostheses provide a reliable solution for restoring mandibular function and alleviating chronic pain. Although numerous clinical studies have reported favorable outcomes, no consensus exists on an optimal prosthetic design that fully replicates the complex anatomy and biomechanics of the native joint [3,4]. As a result, ongoing research continues to refine prosthetic designs, improving biomechanical performance, reducing complications, and enhancing long-term survival [5].

The masticatory system represents a highly complex biomechanical unit, involving dynamic interactions among muscles, bones, ligaments, and joints. Due to the anatomical variability and limited accessibility of the TMJ region, in vivo biomechanical evaluation remains a challenging task. Since such testing in humans or animals is often impractical, mathematical modeling approaches are frequently employed as alternatives [6]. Finite element analysis (FEA) has therefore become a valuable method, enabling simulation of stress and strain distributions in three-dimensional models under physiological loading conditions [7]. In this method, the structure is subdivided into a finite number of elements connected by nodes, allowing stress and displacement patterns to be calculated based on their relative positions [8]. This approach offers distinct advantages in evaluating the mechanical behavior of TMJ prostheses across various functional scenarios. In recent years, the number of studies employing FEA to examine mandibular stress distributions under different functional positions has increased [6,7,9,10]. As highlighted by Allena and Rémond, the emerging field of theramechanics emphasizes how mechanical analysis can guide the development of innovative medical treatments, which aligns with the present study’s use of finite element methods to optimize temporomandibular joint prosthesis design [11].

Material selection plays a crucial role in the long-term success of TMJ prostheses. Among the commonly used alloys, Ti6Al4V and CoCrMo are frequently employed due to their favorable strength, corrosion resistance, and biocompatibility [12]. These materials, however, differ in elastic modulus, fatigue strength, and wear resistance, factors that directly affect the mechanical response of prosthetic components under functional mandibular loads. Moreover, the long-term success of joint replacements is influenced not only by prosthetic material selection but also by the adaptive response of bone tissue, as described by Giorgio et al. where Wolff’s law provides a mechanistic framework explaining how mechanical loading drives bone remodeling and structural evolution over time [13]. Although several studies have examined the mechanical behavior of TMJ prostheses, direct, patient-specific comparisons of Ti6Al4V and CoCrMo under identical anatomical and loading conditions remain relatively scarce, partly because such systems are inherently multi-component constructs that require consideration of complex material interactions [12,14,15]. In this study, linear elastic, homogeneous, and isotropic material properties were assumed for all prosthetic components to reflect typical mechanical behavior under functional masticatory loading conditions. Ti6Al4V and CoCrMo were selected due to their well-established mechanical strength, corrosion resistance, and clinical reliability in load-bearing applications, while Ultra-High Molecular Weight Polyethylene (UHMWPE) was modeled as the fossa component material because of its low friction coefficient and wear resistance in articulating interfaces [14,16,17].

The aim of this study was to compare the biomechanical behavior of patient-specific TMJ prostheses fabricated from Ti6Al4V and CoCrMo alloys using finite element analysis, with a focus on stress distribution, deformation patterns, and areas of potential mechanical failure, offering new insights into the material-dependent mechanical behavior of patient-specific TMJ prostheses based on a direct comparative analysis under identical anatomical and loading conditions.

## 2. Materials and Methods

This finite element analysis study was conducted at the Bahcesehir University School of Dental Medicine, Department of Oral and Maxillofacial Surgery. The study was conducted in accordance with the Declaration of Helsinki and was approved by the Bahcesehir University Non-Interventional Clinical Research Ethics Committee (Approval ID: 2025-02/01; Date: 5 February 2025).

### 2.1. Study Design

There were two study groups, each consisting of a separate three-dimensional mandibular model incorporating a custom-made, right-sided total temporomandibular joint prosthesis fabricated from different biomaterials. In Model 1, the prosthesis was simulated using Ti6Al4V alloy, whereas in Model 2, the same prosthetic design was created using CoCrMo alloy.

### 2.2. Data Collection and Model Construction

**Figure 1:**
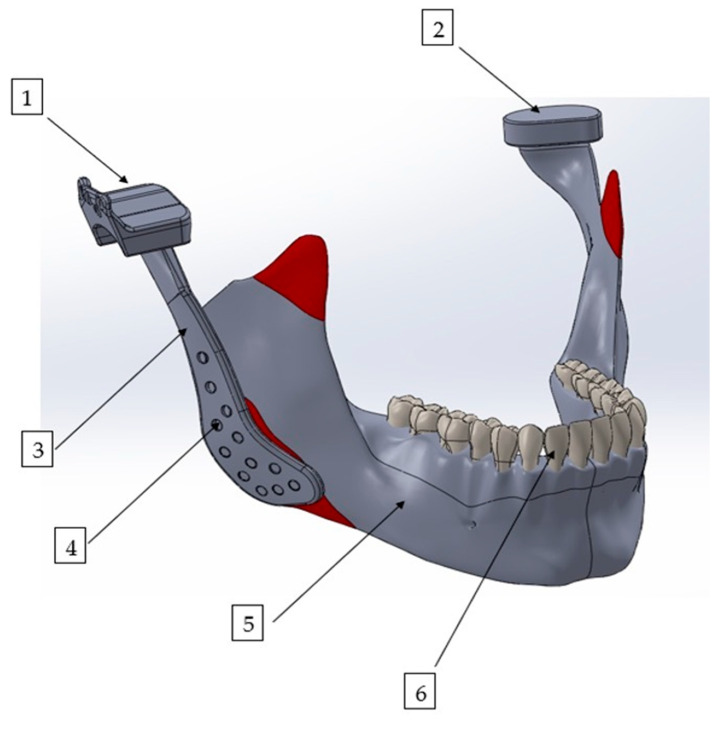
Three-dimensional finite element model of the mandible with a patient-specific TMJ prosthesis. The model illustrates the main components of the reconstruction: (1) fossa prosthesis, (2) TMJ disk, (3) mandibular prosthesis, (4) screws, (5) mandible, and (6) teeth.

Finite element analysis was performed using ANSYS Workbench 2022 R1 (Ansys Inc., Canonsburg, PA, USA). For modeling of the bone structures, computed tomography scans of a healthy patient from our institutional archives were used, which were acquired using a NewTom VGi evo CBCT unit (Cefla S.C., Imola, Italy) operating at 98 kV and 8 mA, with a voxel resolution of 0.2 mm and a field of view (FOV) of 24 cm × 19 cm. The three-dimensional mandibular model was then refined using the Zygote Solid 3D Model (Zygote Media Group Inc., Provo, UT, USA), which is widely recognized for its high anatomical accuracy and resolution, making it suitable for complex biomechanical simulations. The custom-designed total temporomandibular joint prosthesis was modeled using SolidWorks 2012 (Dassault Systèmes, SolidWorks Corp., Waltham, MA, USA), a computer-aided design (CAD) software. The final integrated model of the mandible and the TMJ prosthesis is presented in Figure 1. All model reconstruction and finite element simulations were performed by an experienced engineer (Y.D.) specialized in computational biomechanics.

**Figure 2:**
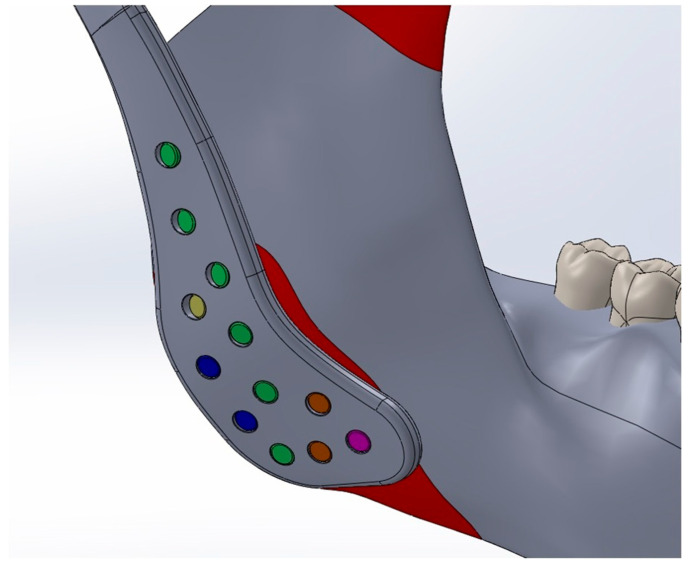
Titanium cortical screws (Ø2.7 mm) of different lengths, represented by different colors, positioned according to anatomical location and standard surgical protocols.

The Young’s modulus and Poisson’s ratio of the biomaterials and anatomical structures were assigned according to values reported in previous studies and are summarized in Table 1 [10,18,19,20,21]. Cortical bone forms the outer surface of both the mandible and maxilla, while cancellous bone occupies the internal regions. However, due to the geometric complexity of modeling layered bone structures and the marked difference in stiffness between bone and the articular disk (approximately 15,000 MPa vs. 44.1 MPa), the potential influence of this variation on stress distribution was considered negligible [22]. This simplification is consistent with previous validated studies and does not substantially affect global stress transfer patterns [23,24,25]. Consequently, only cortical bone material properties were applied in the finite element analysis, and the cancellous component was not modeled separately. To replicate clinical fixation conditions, all fixation screws were modeled as titanium cortical screws with a diameter of 2.7 mm. Multiple screw lengths were used according to their anatomical placement: green screws (2.7 × 6.5 mm), yellow screws (2.7 × 5 mm), blue screws (2.7 × 5.5 mm), orange screws (2.7 × 8.5 mm), and a magenta screw (2.7 × 10.5 mm). The screws were positioned according to standard surgical protocols. (Figure 2)

### 2.3. Boundary Conditions

In the finite element model, boundary conditions were defined by assigning various contact interactions between components to simulate realistic biomechanical behavior. A frictional contact was established between both mandibular prosthesis and the fossa component (UHMWPE), with a coefficient of friction set at 0.05 [26]. A lower frictional coefficient of 0.001 was applied for the contacts between the mandibular condyle and the TMJ disk, as well as between the cranial base and the TMJ disk [27]. To simulate ideal mechanical decoupling, a frictionless contact was defined between both mandibular prosthesis and the native mandible, despite the nominal coefficient of 0.05. Bonded contacts were assigned between the mandibular prosthesis and the fixation screws, and between the screws and the mandible, to ensure rigid fixation and eliminate relative motion at these interfaces. To simulate the absence of direct load transfer between the mandibular prosthesis and the native bone surface, a frictionless contact was defined at their interface, while rigid fixation and load transmission were modeled through bonded screw–bone interactions [26].

To ensure consistency and comparability between the two finite element models, the same meshing strategy and element types were applied in both models. A body sizing approach with quadratic element structures was used for the mandibular prosthesis components (both Ti6Al4V and CoCrMo), TMJ disk, and mandible to improve stress distribution accuracy. In both the Ti6Al4V and CoCrMo mandibular prostheses, a body sizing element size of 0.5 mm was applied, resulting in a mesh consisting of approximately 301,641 nodes and 207,076 elements. The TMJ disk was meshed with a body sizing element size of 1 mm, generating approximately 126,430 nodes and 35,508 elements. The mandibular fossa component was discretized using a hex-dominant meshing method, while the mandible was meshed with a quadratic element structure and an element size of 1.5 mm. This meshing configuration provided a sufficiently fine resolution to accurately capture localized stress concentrations and deformation behavior, while maintaining computational efficiency and ensuring a reliable comparative analysis of the two prosthetic materials.

### 2.4. Loading Conditions

**Figure 3:**
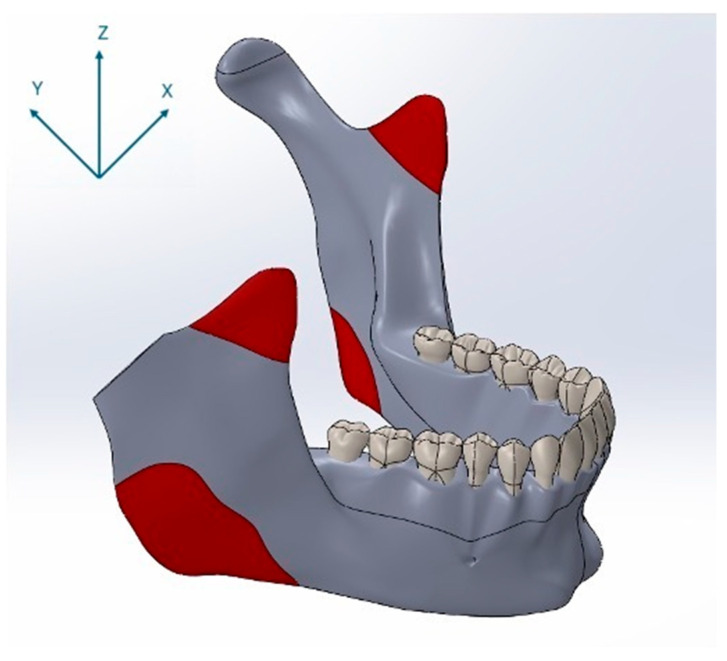
Anatomical muscle attachment sites used as force application points in the finite element model, shown together with the coordinate system (X, Y, Z), indicating load vector orientation.

For the finite element simulations, forces were applied to the three-dimensional mandibular models in the direction of mandibular closure through anatomically defined muscle attachment sites. The locations of these muscle insertion points were determined based on a cadaveric study [28]. Specifically, forces were applied at the anatomical insertion points of the temporalis, masseter, and medial pterygoid muscles. The positions of these muscle attachments on the model are illustrated in Figure 3. The magnitudes and directional components of the masticatory muscle forces applied to the mandible during closure were derived from previously published studies and are presented in Table 2 [26,28,29]. In this study, nominal bite forces corresponding to 50% of reported maximum voluntary bite forces were applied to reflect physiologically relevant loading during normal mastication rather than extreme peak forces [26].

### 2.5. Simulation Procedure

**Figure 4:**
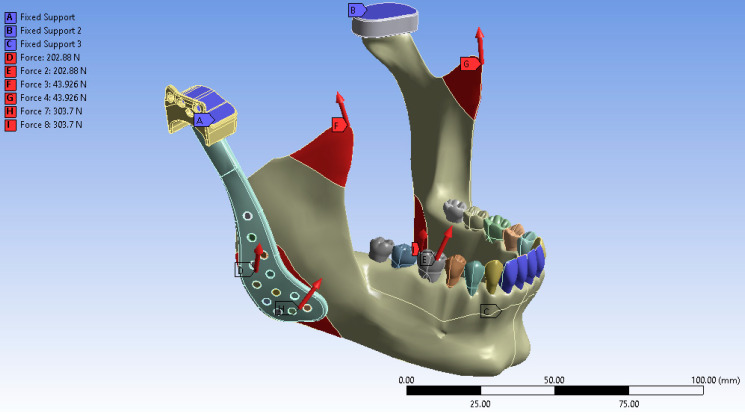
Boundary conditions of the finite element model showing fixation at the anterior teeth, superior surface of the fossa prosthesis, and cranial base to simulate incisal loading.

Following the completion of the modeling process and the definition of muscle force application points, the anterior teeth, the superior surface of the fossa prosthesis, and the cranial base were constrained to replicate an anterior biting condition. In the virtual environment, the anterior teeth, the superior surface of the fossa prosthesis, and the cranial base were fixed to simulate functional loading during incisal loading. The finite element analysis was subsequently performed under these defined boundaries and loading conditions. The final simulated model is illustrated in Figure 4.

As the stress values obtained from finite element analysis are derived from deterministic mathematical computations without variability, statistical analyses were not applicable.

## 3. Results

**Figure 5:**
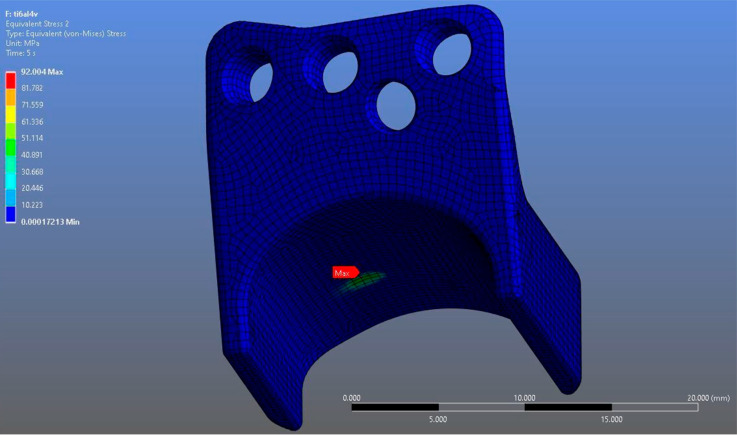
von Mises stress distribution on the fossa prosthesis in the Ti6Al4V model, with a maximum stress value of 92.004 MPa.

**Figure 6:**
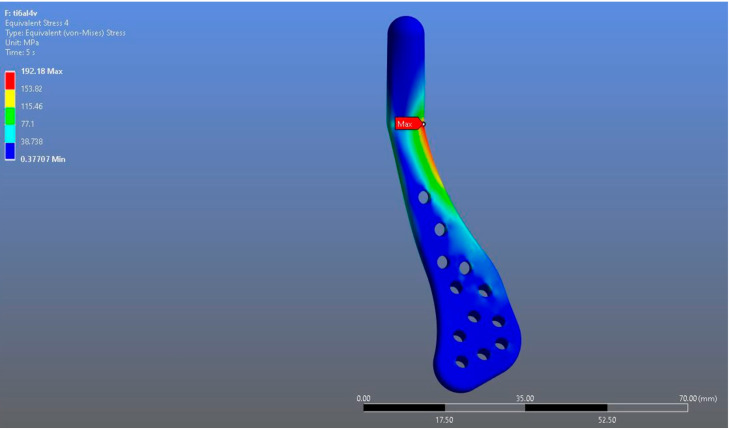
von Mises stress distribution on the mandibular prosthesis in the Ti6Al4V model, with a maximum stress value of 192.18 MPa.

**Figure 7:**
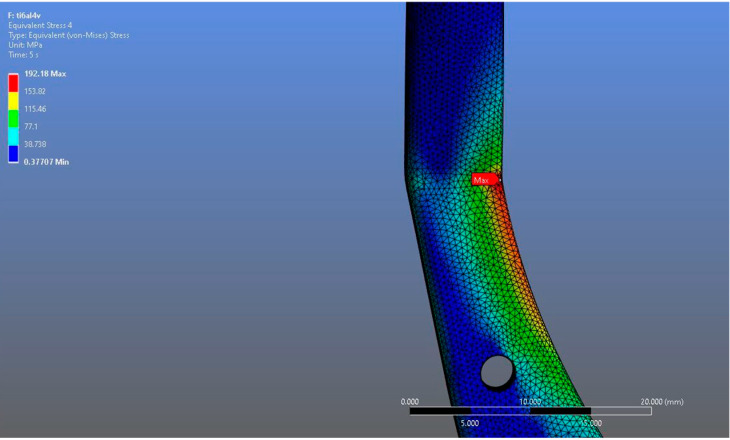
Magnified view of the von Mises stress distribution in the Ti6Al4V model, highlighting localized stress concentration areas.

In Model 1, which incorporated a right-sided patient-specific total temporomandibular joint prosthesis with a mandibular component simulated from Ti6Al4V ELI alloy in contact with a UHMWPE fossa component, the maximum von Mises stress value measured on the fossa prosthesis was 92.004 MPa (Figure 5). Within the same model, the Ti6Al4V mandibular prosthesis component exhibited a maximum von Mises stress of 192.18 MPa (Figure 6 and Figure 7).

**Figure 8:**
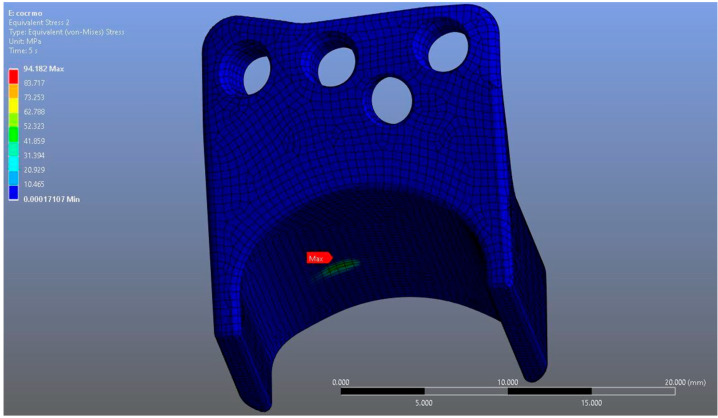
von Mises stress distribution on the fossa prosthesis in the CoCrMo model, with a maximum stress value of 94.182 MPa.

**Figure 9:**
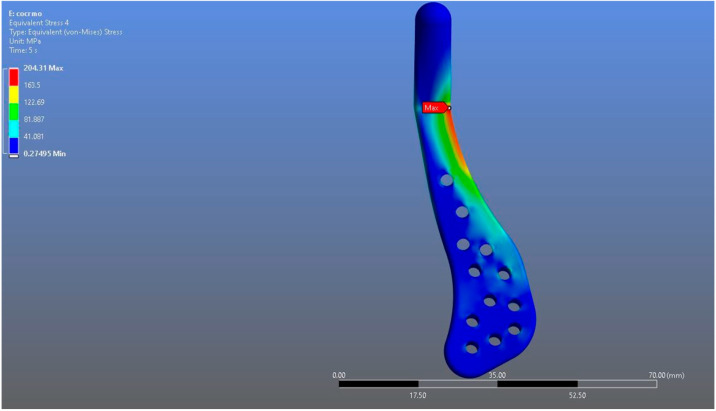
von Mises stress distribution on the mandibular prosthesis in the CoCrMo model, with a maximum stress value of 204.31 MPa.

**Figure 10:**
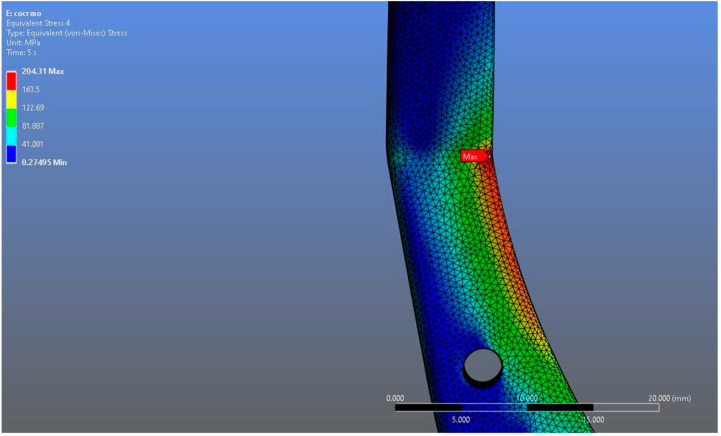
Magnified view of the von Mises stress distribution in the CoCrMo model, highlighting localized stress concentration areas.

In Model 2, which employed an identical prosthesis design simulated from CoCrMo alloy, the maximum von Mises stress observed on the fossa prosthesis was slightly higher, reaching 94.182 MPa, while the mandibular prosthesis demonstrated a maximum von Mises stress value of 204.31 MPa (Figure 8, Figure 9 and Figure 10).

Both models demonstrated stress concentrations in anatomically and biomechanically consistent regions, particularly around the articular interfaces and fixation areas. However, despite the similarity in distribution patterns, numerical differences in stress magnitudes were observed between the two materials under the same loading conditions. It was observed that the maximum stress on the Ti6Al4V prosthesis was lower than that on the CoCrMo prosthesis. When the fossa prosthesis was examined, it was determined that the maximum stress created by the CoCrMo prosthesis against the fossa prosthesis was higher than the stress created by the Ti6Al4V prosthesis.

## 4. Discussion

This study aimed to compare the biomechanical performance of patient-specific temporomandibular joint prostheses fabricated from Ti6Al4V and CoCrMo alloys under identical anatomical and loading conditions using finite element analysis. As material properties play a decisive role in the long-term mechanical stability and structural safety of load-bearing prosthetic devices, understanding how different alloys respond to physiological forces is essential for optimizing prosthesis design [14]. Importantly, the metallic alloys investigated in this study (Ti6Al4V and CoCrMo) are widely used as structural components in load-bearing maxillofacial joint prostheses, while UHMWPE is typically employed as the articulating counter-surface. This combination represents the standard material pairing in current clinical practice, although the metallic components and UHMWPE do not directly contact each other under static conditions, they function together as part of the same load-bearing system. Therefore, analyzing the biomechanical behavior of Ti6Al4V and CoCrMo within this context provides clinically relevant insights into how material selection influences overall prosthesis performance and long-term durability [16,17,30]. By combining patient-derived imaging data with computational modeling, this study contributes to the growing body of work applying finite element analysis to evaluate the mechanical behavior of biomedical alloys in complex joint systems.

The results showed that both Ti6Al4V and CoCrMo prostheses produced stress concentrations in anatomically consistent regions, particularly around the glenoid fossa and ramus, which are known to be critical load-bearing zones during mandibular closure [22,26]. The Ti6Al4V prosthesis demonstrated lower maximum von Mises stresses in both the mandibular (192.18 MPa) and fossa components (92.004 MPa) compared to the CoCrMo prosthesis (204.31 MPa and 94.182 MPa, respectively). These results clearly indicate that alloy selection has a direct impact on stress transfer patterns within the prosthesis and its articulating counterparts, which may have implications for the mechanical stability and service life of the prosthesis under long-term loading [31]. Although the maximum stress values observed in both alloys remained well below their respective fracture strengths, this finding should not be underestimated from either a biomechanical or clinical perspective, since even minor differences between the two alloys could have meaningful implications over time. Even when absolute stress magnitudes are subcritical, their spatial distribution and localized concentration zones can have significant long-term consequences. Repetitive masticatory loading may cause these stress concentrations—even far below the yield strength—to act as initiation sites for microcracks, potentially leading to fatigue-related damage and compromised prosthesis longevity. Moreover, small differences in load transfer patterns can alter the mechanical stimuli to surrounding bone, potentially modifying remodeling dynamics in accordance with Wolff’s law and increasing the risk of stress shielding or aseptic loosening over time. Differential stress fields at the alloy–bone and alloy–polymer interfaces may also accelerate wear, promote micromotion, and generate particulate debris, which in turn can induce peri-implant inflammation and osteolysis [14,31].

From a clinical standpoint, the more favorable stress profile observed in the Ti6Al4V model may therefore provide meaningful advantages for the long-term performance of patient-specific TMJ prostheses. Reduced peak stresses at the bone–implant interface could decrease the likelihood of micromotion, implant loosening, or progressive bone resorption under prolonged functional loading. Furthermore, a lower stress environment on the articular surfaces—including the UHMWPE component—may reduce wear particle generation and contribute to a more physiological load transfer, thereby preserving peri-implant bone quality. Collectively, these biomechanical and biological considerations indicate that even modest differences in stress distribution between alloys can translate into clinically relevant outcomes, underscoring the importance of material selection for the long-term stability and durability of TMJ prostheses [32,33,34]. From a translational standpoint, the biomechanical differences identified in this study highlight how material selection can directly influence clinical outcomes in TMJ reconstruction. Beyond the purely mechanical findings, the lower stress magnitudes observed in Ti6Al4V may translate into better long-term implant integration, lower wear of articulating components, and improved prosthesis longevity. These aspects are particularly relevant for patients with high functional demands or reduced bone density, where minimizing interfacial stress is crucial for maintaining osseointegration and joint stability. Consequently, the choice between Ti6Al4V and CoCrMo should not only consider strength and wear resistance but also the alloy’s ability to achieve mechanical compatibility with surrounding bone, which ultimately determines long-term functional success.

The more favorable stress profile of Ti6Al4V can be explained by its lower elastic modulus compared to CoCrMo. This lower stiffness allows greater energy absorption and facilitates a more homogeneous load transfer, thereby reducing localized stress concentrations within the prosthesis and at the interfaces with bone and polymeric components. In contrast, the substantially higher modulus of CoCrMo results in reduced deformation capacity and greater stress accumulation, which may increase the risk of fatigue-related failure or microdamage during cyclic loading [31,35,36]. It should be noted that, in this study, the contact between the fixation screws and the mandibular bone was modeled as bonded to simplify the analysis and isolate the effect of material properties on global stress distribution [26]. This approach intentionally eliminates potential micromotion at the bone–implant interface, which, although clinically relevant, was beyond the scope of the current investigation. Future studies incorporating frictional or nonlinear contact conditions could provide further insight into how such micromechanical interactions influence long-term stability and fatigue performance. Notably, the lower stress transmission to the fossa component in the Ti6Al4V model may also reduce the risk of wear and mechanical degradation of the UHMWPE surface, a factor crucial for the long-term structural integrity of joint replacement systems [33]. The differences in stress response observed between these alloys thus highlight the importance of modulus matching with surrounding biological tissues as a key design parameter.

The stress distribution patterns predicted in this study are consistent with findings from previous experimental and cadaveric investigations of mandibular biomechanics and TMJ prosthesis performance [32,37]. In vitro strain-gauge analyses have demonstrated that occlusal loading leads to concentrated stresses around the condylar process and fixation regions, aligning with our observation of peak stress localization in these anatomically critical zones and with the strain-shielding and load transfer patterns reported in cadaveric models. Similarly, finite element analysis validated against experimental measurements have reported comparable stress distributions under various functional loading scenarios, despite differences in geometry and boundary conditions [37]. Moreover, comparative analyses have shown that stress magnitudes predicted by finite element models closely match experimental measurements, supporting the validity of the present results. Tribological investigations further demonstrate that UHMWPE–metal bearing pairs exhibit contact-condition-dependent stress and wear behavior, with CoCrMo interfaces generating higher local stresses than Ti6Al4V, resulting in increased wear particle generation and potential biological responses [32]. Collectively, the convergence of anatomical stress locations, order-of-magnitude agreement in measured versus simulated values, and consistency in tribological behavior across studies qualitatively supports the predictive accuracy of the present finite element analysis.

Evidence from orthopedic prosthesis research further supports these interpretations. In hip and knee replacements, CoCrMo has been extensively used as an articulating material due to its superior wear resistance and fatigue performance. However, the release of cobalt and chromium ions due to wear and corrosion has been identified as a potential drawback, associated with local adverse reactions and systemic exposure. Ti6Al4V, on the other hand, combines a lower elastic modulus—closer to that of cortical bone—with a stable oxide layer that significantly limits ion release, thereby offering enhanced long-term biocompatibility and mechanical compatibility. Meanwhile, UHMWPE continues to serve as the material of choice for counterfaces in both orthopedic and maxillofacial prostheses due to its low friction coefficient and high wear resistance. However, challenges such as creep deformation and oxidative degradation persist. These established orthopedic experiences provide an important translational framework, reinforcing the relevance of the present findings and emphasizing the engineering considerations that must balance biomechanical performance with wear resistance in alloy selection for TMJ prostheses [38,39,40,41,42,43].

Finite element analysis proved to be a powerful methodology in this context. The TMJ is among the most complex joints in the human body, and anatomical constraints limit direct experimental testing of its biomechanical behavior. FEA offers a reproducible, non-invasive framework for simulating realistic loading conditions, quantifying stress distributions, and evaluating the performance of biomaterials under physiologically relevant forces [38,39]. By integrating patient-derived CT data into the modeling, the present study enhanced both the anatomical fidelity and the translational accuracy of the simulation. Similar computational approaches have been widely applied in orthopedic and maxillofacial biomechanics, demonstrating their value in guiding material selection and prosthesis optimization [40,41,44,45,46,47].

Another important aspect that should be considered when interpreting the present findings is the potential impact of inter-patient variability on stress distribution. Mandibular anatomy can vary markedly among individuals—including differences in cortical bone thickness, ramus length and inclination, and overall mandibular geometry—and such morphological variability has been shown to significantly influence both the magnitude and distribution of stresses in finite element models. These anatomical differences can alter how mechanical loads are transferred through the prosthesis and surrounding bone structures, potentially shifting regions of stress concentration or changing load transfer pathways [48]. For example, variations such as reduced cortical thickness or a more vertically oriented ramus may increase local stresses at the prosthesis–bone interface, whereas a broader ramus or deeper fossa may promote a more homogeneous stress distribution. Because the present finite element model was based on a single patient-specific geometry, the results reflect one possible biomechanical scenario rather than the full range of clinical variability [49]. Future studies should therefore incorporate parametric modeling strategies and population-specific anatomical datasets to capture the spectrum of interindividual differences and to improve the generalizability and predictive power of finite element analyses.

Nevertheless, several limitations must be recognized. This analysis was based on a single patient-specific model, which limits generalizability. Only static loading conditions were considered, whereas real-life joint function involves dynamic and cyclic forces. Material properties were treated as homogeneous and isotropic, which does not fully reflect the anisotropy of cortical bone and the viscoelasticity of soft tissues. In addition, stress distribution within the fixation screws and screw–bone interfaces were not evaluated. This decision was made to reduce computational complexity, as high-resolution meshing of screw geometries significantly increases simulation demand. Since the primary aim was a comparative analysis of prosthetic alloys, screws were modeled solely for stability. Furthermore, no statistical validation was performed due to the deterministic nature of finite element analysis, which, while ensuring computational repeatability, limits the ability to account for inter-patient variability and reduces the predictive capacity of the model for real-world clinical outcomes. Future investigations should incorporate dynamic loading cycles, fatigue simulations, multiphysics models that consider wear and temperature, and larger patient datasets to improve predictive accuracy. Moreover, detailed evaluation of fixation screw mechanics will be essential to better assess the risk of loosening and long-term stability. Expanding the analysis to a broader patient population and integrating more physiologically relevant loading conditions would also enhance the robustness and translational relevance of future finite element studies.

In conclusion, this finite element analysis demonstrated that Ti6Al4V and CoCrMo alloys, both widely accepted as standard biomaterials in load-bearing prostheses, exhibit distinct biomechanical profiles when applied in patient-specific TMJ models. Ti6Al4V showed lower stress magnitudes and more favorable load distribution, while CoCrMo provided higher wear resistance and endurance. These findings emphasize the critical role of alloy selection in prosthetic design and highlight finite element analysis as an effective engineering tool for optimizing mechanical performance in next-generation patient-specific TMJ prostheses. Future studies should aim to include larger patient cohorts, incorporate dynamic and fatigue loading scenarios, and refine material models to more accurately reflect biological complexity, thereby increasing the clinical applicability and predictive value of finite element simulations.

## Figures and Tables

**Table 1:** Young’s modulus and Poisson ratios of the materials used in modeling.

	Young’s Modulus (mPa)	Poisson’s Ratio
Disk	44.1	0.35
Cortical Bone	15,000	0.3
Ti6Al4V	110,000	0.33
CoCrMo	220,000	0.3
UHMWPE	1170	0.4

**Table 2:** Forces and force components of the masticatory muscles closing the mandible.

MUSCLE	Fx (N)	Fy (N)	Fz (N)
Temporalis	−8.435	10.91	41.705
Medial Pterygoid	147.525	−113.225	240.11
Masseter	−60.91	−54.175	185.785

## Data Availability

The raw data supporting the conclusions of this article will be made available by the authors on request.

## Abbreviations

The following abbreviations are used in this manuscript:

TMJ	Temporomandibular joint
FEA	Finite element analysis
CAD	Computer-aided design
